# The Effect of the *Lacticaseibacillus paracasei* BEPC22 and *Lactiplantibacillus plantarum* BELP53 Combination (BN-202M) on Body Fat Percentage Loss in Overweight Individuals: A Randomized, Double-Blind, Placebo-Controlled Study

**DOI:** 10.3390/nu16131993

**Published:** 2024-06-23

**Authors:** Han-Seul Kwon, Seok-Jin Kim, Kum-Joo Shin, Sanghoon Kim, Jongbok Yun, Jaewoong Bae, Hyun-Ji Tak, Na-Rae Lee, Hyeong-Jun Kim

**Affiliations:** 1 Department of Korean Obstetrics and Gynecology, College of Korean Medicine, Semyung University, Jecheon 27136, Republic of Korea; kwonhansel@naver.com; 2 R&D Center, Hecto Healthcare Co., Ltd., Seoul 06142, Republic of Koreajb@hecto.co.kr (J.Y.);; 3 Department of Bioscience and Biotechnology, Konkuk University, Seoul 05029, Republic of Korea; 4 Research Institute for Bioactive-Metabolome Network, Konkuk University, Seoul 05029, Republic of Korea

**Keywords:** *Lacticaseibacillus paracasei*, *Lactiplantibacillus plantarum*, obesity, overweight, body fat percentage, probiotics, gut microbiota

## Abstract

BN-202M is derived from humans and consists of two strains, *Lacticaseibacillus paracasei* BEPC22 and *Lactiplantibacillus plantarum* BELP53. Body fat reduction effect and safety of BN-202M were assessed in overweight participants. A total of 150 participants were randomly assigned to the BN-202M and placebo groups at a 1:1 ratio. Dual-energy X-ray absorptiometry was used to objectively measure body fat. After 12 weeks of oral administration, the body fat percentage (−0.10 ± 1.32% vs. 0.48 ± 1.10%; *p* = 0.009) and body fat mass (−0.24 ± 1.19 kg vs. 0.23 ± 1.05 kg; *p* = 0.023) of the BN-202M group decreased significantly compared to those of the placebo group. The body weight (−0.58 kg, *p* = 0.004) and body mass index (BMI; −0.23, *p* = 0.003) was found to decrease significantly at 12 weeks in the BN-202M group, but not in the placebo group. Metabolome analysis revealed that β-alanine, 3-aminoisobutyric acid, glutamic acid, and octopamine decreased in the weight-decreased BN-202M post-intake group. In the gut microbiota analysis, Akkermansia showed a statistically significant increase in the BN-202M group post-intake compared to the placebo group. No serious adverse events were observed in either group. These results suggest that BN-202M is safe and effective for reducing body fat and weight.

## 1. Introduction

Obesity is a condition in which energy intake and consumption are unbalanced, causing excess energy to accumulate as fat and resulting in abnormally increased body fat [1] and various metabolic abnormalities [2]. The body mass index (BMI) has long been used to define obesity in adults. Overweight is defined as BMI 25–29.9 kg/m^2^, and obesity is defined BMI ≥30 kg/m^2^ [3]. Previous studies have revealed an association between high BMI and chronic diseases, such as diabetes mellitus [3], cardiovascular disease [4], obstructive sleep apnea–hypopnea syndrome [5], knee osteoarthritis [6], nonalcoholic fatty liver disease [7], and chronic kidney disease [8]. Obesity contributes to metabolic issues, including insulin resistance and hyperglycemia, and can lead to type 2 diabetes and dyslipidemia, thereby increasing healthcare expenses [9].

In 2022, the Global Metabolic Disorders Therapeutics Market was valued at USD 68.1 billion and is anticipated to considerably expand, achieving a value of USD 122.3 billion by 2030. The market is projected to grow at a compound annual growth rate (CAGR) of 7.8% from 2023 to 2030 [10]. With the rising prevalence of obesity and diabetes, there is an urgent need for effective approaches to prevent and manage metabolic disorders. Traditional pharmacological treatments are often associated with side effects and limitations, necessitating the exploration of alternative solutions. In this context, probiotics have emerged as an innovative approach to managing metabolic disorders [11].

Probiotics are live microorganisms that provide health benefits to the host when consumed in adequate quantities. Some studies reported that probiotics positively impact obesity [12] and insulin resistance [13]. Short-chain fatty acids (SCFAs), propionate, and butyrate induce the expression of gluconeogenesis-related genes through various mechanisms and reduce body weight and fat cells by activating gluconeogenesis in the intestines [14]. Additionally, in animal experiments, acetate increased browning of white adipose tissue (WAT) and brown adipose tissue (BAT) with respect to thermogenesis [15].

In our previous study, we reported that a formulation with *Lacticaseibacillus paracasei* BEPC22 and *Lactiplantibacillus plantarum* BELP53 (BN-202M) decreased body weight gain at 6 weeks and decreased the food efficiency ratio, white adipose tissue volume, and adipocytes in high-fat diet-fed mice through adipocyte differentiation inhibition, the suppression of lipid metabolism, and increased lipid oxidation [16].

In this study, we aimed to evaluate the body fat reduction effect of BN-202M in overweight individuals for 12 weeks.

## 2. Materials and Methods

### 2.1. Clinical Trial Design

This single-center, randomized, double-blind, placebo-controlled intervention study was conducted to confirm the efficacy and safety of BN-202M supplementation for body fat loss in obese and overweight participants. In this clinical trial, lifestyle factors, including exercise and eating habits, were consistently maintained throughout the test period.

### 2.2. Preparation of Samples and Treatment

Each 2 g sachet of BN-202M contained a 5 × 10^10^ colony-forming unit (CFU) blend of *Lacticaseibacillus paracasei* BEPC22 and *Lactiplantibacillus plantarum* BELP53. The formulation was mixed with a carrier powder (73.9% maltodextrin, 3% mocha coffee flavor powder, and 0.1% aspartame). A placebo sachet containing only the carrier powder with added colors (gardenia yellow and monascus red) was also manufactured according to Good Manufacturing Practice (GMP) guidelines at a Korean government-approved facility. Both the BN-202M and placebo sachets were dispensed by a research pharmacist. The participants were instructed to consume one sachet daily on an empty stomach for 12 weeks. If a participant dropped out of the study, their measurements were recorded at the time of their final treatment, with additional follow-up assessments conducted within one month. To improve compliance, we reviewed participants’ medication diaries and conducted sachet counts at each visit.

### 2.3. Participants

Participants (aged 19–60 years) were recruited from Jecheon-si and Semyung University Korean Medicine Hospital in the Republic of Korea between January 2022 and May 2023. After the initial screening, 150 participants with a BMI of 25–29.9 kg/m^2^ were selected.

The exclusion criteria were as follows: (1) history of hypersensitivity to raw materials or natural products in this study; (2) weight gain or loss of 5 kg or more within 3 months; (3) excessive alcohol consumption (>20 g/day); (4) diagnosis of dyslipidemia (hyperlipidemia) and cannot stop taking drugs that can affect lipid changes; (5) continuous treatment or medication for more than 10 days due to gastrointestinal disorders (gastric ulcer, chronic digestive disorder, etc.); (6) uncontrolled hypertension (sitting systolic blood pressure (sitSBP) ≥ 160 mmHg or sitting diastolic blood pressure (sitDBP) ≥ 95 mmHg); (7) patients with diabetes who are on insulin or oral hypoglycemic medications; (8) history of surgery, such as satellite type or intestinal resection or other surgical operations to lose weight within 6 months prior to screening; (9) administration of drugs (including herbal medicines or health functional foods) that can affect body weight within 3 months prior to screening; (10) uncontrolled hyperthyroidism or hypothyroidism; (11) clinically significant hematological, cardiac, lung, or neurological disease or other serious systemic disorders; (12) clinically significant renal dysfunction or chronic renal failure; (13) clinically significant liver dysfunction, liver cirrhosis, or chronic liver disease; (14) history of malignancy within 5 years prior to screening; (15) eating disorders, psychotic disorders, including depression, drug addiction; (16) autoimmune diseases; (17) anemia/porphyria; (18) pregnant and breastfeeding women, as well as women of childbearing age who do not utilize suitable contraceptive methods (but cannot take oral contraceptives); (19) participants who have participated or plan to participate in other clinical (human application) trials within 3 months prior to screening; and (20) other inadequate participants assessed by the study investigators.

### 2.4. Randomization

A separate investigator, who was not involved in recruiting participants or collecting data, used a computer-generated randomization code to assign the participants. They were then evenly divided into either the BN-202M or placebo groups at a 1:1 ratio.

### 2.5. Ethics

This study was approved by the Ethics Committee of the Semyung University Korean Medicine Hospital (IRB No. SMJOH-2022-05) and registered in the Korean Clinical Research Information Service (KCT0007268). All participants voluntarily provided written informed consent before enrolment. The investigator detailed the purpose, procedures, and potential results of the study before obtaining signatures. This clinical trial adhered to the ethical and scientific standards set by the Korean Good Clinical Practice (KGCP) and was conducted in accordance with the ethical principles of the 1975 Declaration of Helsinki.

### 2.6. Sample Size

The sample size for this study was based on a previous study that examined the effect of LP28 probiotic supplementation on body fat percentage. The earlier study found that the average reduction in fat percentage was 0.08 in the placebo group and −0.50 in the group receiving LP28 probiotic supplementation [17]. The difference in change of fat percentage between the placebo and LP28 probiotics was −0.58. The estimated standard deviation was 0.93, which was the highest value recorded in the reference test. A type I error rate of 5% and a type II error rate of 10% were anticipated based on previous studies involving the efficacy of LP28 probiotic supplementation. To adhere to the central limit theorem—which posits that the distribution of the sample mean approaches normality when the sample size is 30 or greater—each group in this study required 54 participants. Factoring in an expected dropout rate of 28% for each group, the total number of participants needed was determined to be 150.

### 2.7. Anthropometric and Biochemical Analyses

Efficacy was measured at baseline and 12 weeks, and safety was determined at baseline, 6 weeks, and 12 weeks. Body fat percentage, as the primary endpoint, and body weight, BMI, body fat mass, and lean body mass, as secondary endpoints, were measured using dual-energy X-ray absorptiometry (DEXA) [18]. Waist (center of the navel) and hip (center of the protruding area) circumferences were measured using a tape measure, and the ratio of waist circumference to hip circumference was calculated. In addition, adiponectin, leptin, and insulin levels were collected from the plasma and analyzed. Total cholesterol, high-density lipoprotein (HDL) cholesterol, low-density lipoprotein (LDL) cholesterol, triglycerides, and glucose in the serum were analyzed to assess the safety and efficacy of BN-202M at baseline, 6 weeks, and 12 weeks. Safety was assessed via blood pressure, pulse, and body temperature measurements on every visit as well as via blood laboratory testing, including white blood cell (WBC), red blood cell (RBC), hemoglobin, hematocrit, platelets, albumin, alanine aminotransferase (ALT), aspartate aminotransferase (AST), gamma-glutamyl transferase (γ-GTP), alkaline phosphatase (ALP), blood urea nitrogen (BUN), creatinine, glucose, total bilirubin, triglyceride, total cholesterol, HDL cholesterol, LDL cholesterol, and uric acid.

### 2.8. Metabolome Analysis

To extract metabolites from human feces, 1 mL of methanol containing an internal standard solution (2-chloro-L-phenylalanine, 1 mg/mL in water) was added to approximately 20 mg of feces. The mixture was homogenized using a Retsch MM400 Mixer mill (Retsch GmbH & Co. Haan, Nordhein-Westfalen, Germany) at 30 s^−1^ for 10 min and then sonicated for 10 min. Subsequently, the mixtures were centrifuged at 27,000× *g* for 10 min at 4 °C. The supernatant was filtered using 0.2 μm polytetrafluoroethylene (PTFE) syringe filters (Chromdisc, Daegu, Republic of Korea) and fully dried with a speed vacuum concentrator (Biotron, Seoul, Republic of Korea). The dried samples were used for metabolomic analysis.

GC-TOF-MS analysis was performed using an Agilent 7890A GC system (Agilent Technologies, Palo Alto, CA, USA) equipped with a Pegasus HT TOF-MS system (Leco Corporation, St. Joseph, MI, USA). To separate the metabolites, a TRX-5MS column (30 m length × 0.25 mm i.d. × 0.25 μm particle size; Restek Corp., St. Joseph, MI, USA) was used. Helium was used as the carrier gas at a constant flow rate of 1.5 mL/min. One microliter of each derivatized sample was injected into a 15:1 split. The analytical conditions followed those reported in previous reports [16,19].

For metabolome data processing, the raw data from the GC-TOF-MS analysis were converted into NetCDF (*. cdf) using the LECO ChromaTOF. Peak detection, retention time correction, and alignment were performed using MetAlign software Version 041012 (http://www.metalign.nl). Metabolites were tentatively identified by comparing the retention times and fragmentation patterns of standard compounds under identical conditions or by using available databases, such as the National Institutes of Standards and Technology Library (NIST; version 2.0, 2011; FairCom, Gaithersburg, MD, USA). Multivariate statistical analyses were conducted using SIMCA-P+ software (version 15.0.2; Umetrics, Umea, Sweden). Significantly discriminant metabolites were selected according to their variable importance in the projection (VIP) value >1.0, based on a partial least squares-discriminant analysis (PLS-DA) score plot, and significant differences (*p* < 0.05) were tested by one-way analysis of variance (ANOVA) using STATISTICA (version 7.0, StaSoft Inc., Tulsa, OK, USA).

### 2.9. Gut Microbiota Analysis of Fecal Samples Using Next-Generation Sequencing (NGS)

The fecal samples were stored at −80 °C until DNA extraction. Genomic DNA extraction from the total bacterial content was performed using the Maxwell^®^ RSC PureFood GMO and Authentication Kit (Promega, Madison, WI, USA) following the manufacturer’s instructions. The DNA concentration was determined using a UV-vis spectrophotometer (NanoDrop 2000c, ThermoFisher Scientific, Waltham, MA USA), and quantification was conducted using the QuantiFluor^®^ ONE dsDNA System (Promega, Madison, WI, USA). The extracted DNA samples were stored at –20 °C until further experimentation.

For gut microbiota analysis, the V3–V4 variable region of the 16S rRNA gene was amplified from the DNA extracts using the Illumina 16S metagenomic sequencing library protocol. Two PCRs were performed on the template DNA. Initially, the DNA was amplified using primers specific to the V3–V4 region of the 16S rRNA gene, incorporating the Illumina overhang adaptor. Each PCR reaction contained a DNA template (~10–12 ng), forward primer (1 μM), reverse primer (1 μM), 2X Kapa HiFi Hotstart ready mix (Kapa Biosystems, Wilmington, MA, USA), and PCR-grade water. The PCR amplification consisted of an initial denaturation step at 95 °C for 3 min, followed by 25 cycles of denaturation at 95 °C for 30 s, annealing at 55 °C for 30 s, extension at 72 °C for 30 s, and a final extension step at 72 °C for 5 min. The PCR products were visualized using gel electrophoresis. Successful products were purified using AMPure XP magnetic bead-based purification (Beckman Coulter, Brea, CA, USA) and run on an Agilent Bioanalyzer for quality analysis. A second PCR was conducted to index each sample using Illumina Nextera XT indexing primers. The purified DNA was subjected to 8 amplification cycles. Successful products were repurified and quantified using the QuantiFluor^®^ ONE dsDNA System, Promega, Madison, WI, USA. The sample pool (4 nM) was denatured with 0.2 N NaOH, then diluted to 20 pM, and combined with 10% (*v*/*v*) denatured 8 pM PhiX, prepared following the Illumina guidelines. Sequencing was performed on a MiSeq platform using a 2 × 300 cycle V3 kit, following the standard Illumina protocols.

Gut microbiota analysis was conducted using the QIIME 2 2023.05 pipeline. Paired-end sequence data were demultiplexed and joined using the MiSeq Reporter and q2-vsearch plugin, respectively. Merged sequences were quality-filtered using the q2-quality-filter plugin and denoised with Deblur (via q2-deblur). All amplicon sequence variants (ASVs) were aligned with mafft (via q2-alignment). Alpha diversity metrics (Observed ASVs, Shannon, Simpson, and Faith PD) and beta diversity metrics (Bray–Curtis dissimilarity) were estimated using q2-diversity after subsampling without replacement. Taxonomy was assigned to the ASVs using the classify-sklearn method of the q2-feature-classifier plugin with default parameters against the SILVA DB v138.1. Permutational multivariate analysis of variance (PERMANOVA) was used to determine significant differences in the bacterial structure. Linear discriminant analysis effect size (LEfSe) was conducted to detect significant differences in the bacterial taxa (LDA score > 3.0).

### 2.10. Statistical Analysis

To evaluate the statistical significance of the differences between groups, we used the independent two-sample t-test or Mann–Whitney U test for continuous variables, along with Pearson’s chi-squared test or Fisher’s exact test for categorical variables. Differences within each group were assessed using the paired t-test or Wilcoxon signed-rank test for continuous data and McNemar’s test or exact McNemar’s test for categorical data. All statistical tests were conducted using the SAS software (Version 9.4). A *p*-value below 0.05 was deemed significant, whereas a *p*-value above 0.05 was considered nonsignificant. The analysis of primary and secondary endpoints was based on the per-protocol (PP) approach, which included participants who fully adhered to the study protocol without major deviations. Safety assessments were performed in an intention-to-treat (ITT) population comprising all participants who had consumed at least one dose of the investigational product.

## 3. Results

### 3.1. Demographic Characteristic

**Figure 1:**
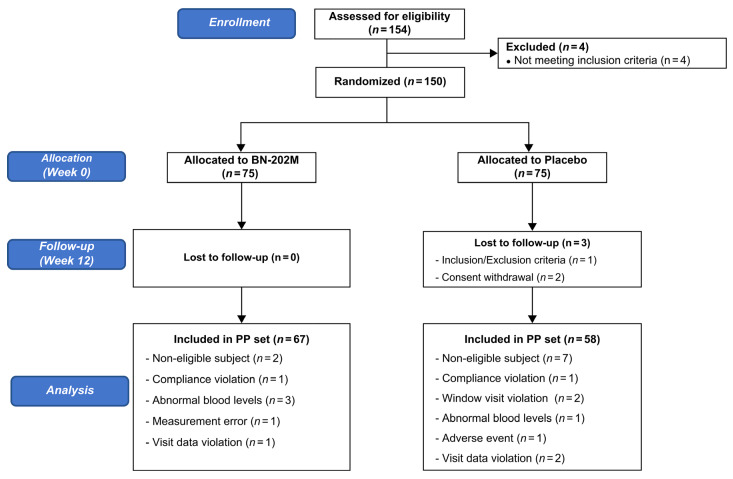
Flow chart of the registered participants for the clinical trial (PP: per protocol). Adverse reactions not related to investigational product: tuberculosis (*n* = 1).

Screening was conducted on 154 participants, and 150 participants were randomly assigned, excluding 4 participants based on the exclusion criteria. Accordingly, 75 participants were assigned to each group. Of these, 149 participants (75 in the BN-202M group and 74 in the placebo group) were included in the intention-to-treat (ITT) group, excluding 1 subject who withdrew consent. Over the course of the trial, 25 participants were excluded for reasons such as dropping out, withdrawal of consent, noncompliance, adverse effects, missed visits, measurement inaccuracies, and abnormal blood values related to diet. Consequently, 125 participants (67 in the BN-202M group and 58 in the placebo group) were included in the per-protocol (PP) group (Figure 1). There were no significant differences in sex, height, weight, BMI, SBP, DBP, pulse rate, body temperature, or current alcohol consumption between the groups, except for age, which showed statistically significant differences (*p* < 0.05; Table 1).

### 3.2. Efficacy and Safety Evaluation

#### 3.2.1. Anthropometric Measurement

The mean of body fat percentage at baseline was 39.62 ± 4.74 in the BN-202M group and 38.06 ± 5.13 in the placebo group, with no statistically significant difference between the two groups. The change in body fat percentage was –0.10 ± 1.32 in the BN-202M group and 0.48 ± 1.10 in the placebo group. The difference in body fat percentage improvement between the two groups was statistically significant (*p* = 0.009). Furthermore, body fat mass (kg) was significantly lower in the BN-202M group than in the placebo group (*p* = 0.023). A statistically significant decrease in body weight (−0.58 ± 1.59, *p* = 0.004), BMI (−0.23 ± 0.60, *p* = 0.003) and hip circumference (−0.23 ± 0.92, *p* = 0.013) change was confirmed only within the test group. No statistical significance was observed in the control group. Between the two groups, there were no significant differences in the remaining secondary endpoints; however, there was a statistically significant difference in the change in serum leptin concentration in the BN-202M group, whereas no statistically significant difference was observed in the placebo group (Table 2).

#### 3.2.2. Safety

Hematological (WBC, RBC, hemoglobin, hematocrit, and platelets) and hematochemistry (albumin, ALT, AST, γ-GT, ALP, BUN, creatinine, glucose, total bilirubin, triglyceride, total cholesterol, HDL cholesterol, LDL cholesterol, and uric acid) markers in the plasma were analyzed for safety. All changes were within the normal range, and none of the changes were judged to be clinically significant by the investigator. There were statistically significant differences in the hematological test results for RBC (*p* < 0.001), hemoglobin (*p* < 0.001), and hematocrit (*p* < 0.001) in the within-group comparison of the BN-202M group; there was a statistically significant difference in hemoglobin (*p* = 0.041) in the within-group comparison of the placebo group. The blood chemistry test results show a statistically significant difference in the uric acid levels between the groups (*p* = 0.006). There were statistically significant differences in the total bilirubin (*p* = 0.023) and uric acid (*p* = 0.028) in the within-group comparison of the BN-202M group, and in the ALP (*p* = 0.005), ALT (*p* = 0.018), HDL cholesterol (*p* = 0.039), and LDL cholesterol (*p* = 0.022) in the within-group comparison of the placebo group (Table 3). The most common adverse events were infections and infestations, with one case each of COVID-19, enteritis, nasopharyngitis, periodontitis, tonsillitis, and upper respiratory tract infection in the BN-202M group and three cases of COVID-19, two cases of enteritis, one case of latent tuberculosis, and one case of otitis externa in the placebo group. In addition, there were cases of musculoskeletal symptoms, such as back pain in the BN-202M group and myalgia in the placebo group. One case of dysmenorrhea was reported only in the BN-202M group, and one case of general symptoms, such as headache, was reported only in the placebo group.

### 3.3. Differences in Gut Metabolome and Microbiome

**Figure 2:**
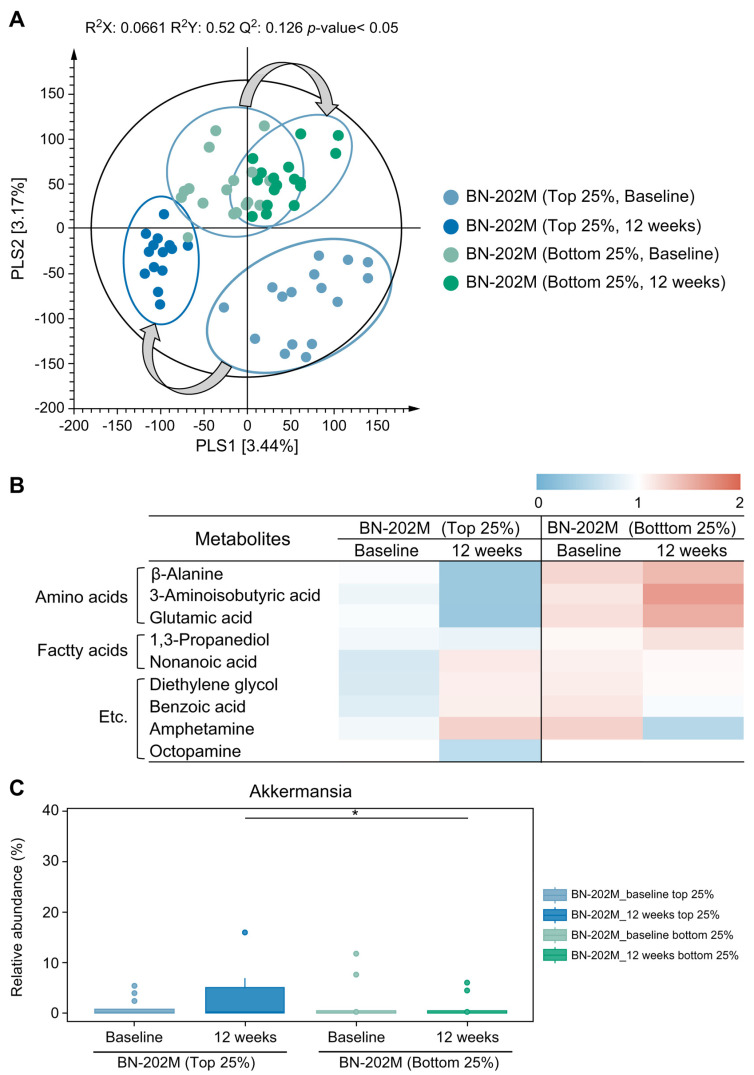
Comparison of gut metabolites and microbiome. (**A**) Partial least squares discriminant analysis (PLS-DA) score plot based on the GC-TOF-MS analysis results. (**B**) Heatmap representing the relative abundance of significantly discriminant metabolites (VIP > 1.0, *p* < 0.05) based on the PLS-DA model. (**C**) Box plot summarizing the significant differences at the genus level for the top and bottom 25% in the baseline and post-intake BN-202M groups.

To identify weight loss-relevant metabolites, we conducted non-targeted metabolome analysis using the top 25% and bottom 25% of body fat loss rates in the BN-202M group baseline and after 12 weeks of intake. The PLS-DA score plot demonstrates obvious distinctions between the top 25% and the bottom 25% by PLS2 and shifting pattern (gray arrows) across the PLS1 axis after the intake of the BN-202M (Figure 2A). Statistically significant metabolites were selected and identified based on the PLS-DA score plot, resulting in nine metabolites (Figure 2B). Among the nine metabolites, β-alanine, 3-aminoisobutyric acid, glutamic acid, 1,3-propanediol, and octopamine decreased after 12 weeks of intake of BN-202M in the top 25% group but increased in the bottom 25% group. In contrast, the top 25% bracket of body fat loss showed increases in nonanoic acid, diethylene glycol, benzoic acid, and amphetamine, whereas those of the bottom 25% bracket were diminished.

To assess the impact of BN-202M supplementation on the gut microbiota of the study participants, fecal samples were collected baseline and after 12 weeks of intake in the BN-202M group. When comparing the top and bottom 25% of participants with body fat loss rates in baseline and 12-week intake in the BN-202M group, Akkermansia demonstrated a statistically significant increase in the 12-week intake of the top 25% group compared to that of the 12-week intake of the bottom 25% group (*p* < 0.05; Figure 2C).

## 4. Discussion

Research has shown that *L. plantarum* strains are effective in reducing obesity in mice by activating the PPARα/CPT1α pathway and decreasing the expression of SREBP-1 and tDGAT1 mRNA or by boosting the expression of genes related to bile secretion, such as cholesterol 7α-hydroxylase (Cyp7α1) [20]. Additionally, these strains have been observed to decrease body weight and fat volume in obese mice on a high-fat diet by suppressing lipogenic genes, including PPARγ, HSL, SCD-1, and FAT/CD36 [21]. The *Paracasei* strain has shown weight management potential in mice. *L. paracasei* supplementation was found to reduce weight gain and decrease fat accumulation in the livers of laboratory mice fed a high-fat diet [22]. To create an anti-obesity probiotic, *L. paracasei* BEPC22 and *L. plantarum* BELP53 were chosen because of their combined effects of inhibiting fatty acid absorption and glucose uptake and their capacity to synergistically produce and utilize metabolites. Within the culture medium, BEPC22 produces glyceric acid, which BELP53 utilizes, whereas BELP53 generates malic acid, which BEPC22 utilizes. This suggests a mutualistic relationship between BEPC22 and BELP53 based on the cross-feeding of glyceric and malic acids. An optimal mix of 60% BEPC22 and 40% BELP53 was established as the most effective in reducing adipogenesis through in vitro assays [16]. This complex was named BN-202M. We performed DEXA to confirm the effect of BN-202M on body fat loss. Consistent with the DEXA results, BN-202M supplementation considerably lowered the body fat percentage and body fat mass compared to a placebo. Although no significant differences in body weight or BMI were observed between the two groups, body weight and BMI were markedly reduced in the BN-202M group. This suggests an association between overall body composition improvement and BN-202M supplementation.

Numerous studies have been conducted since the association between the gut microbiota and obesity was established, particularly through investigations involving obese and lean mouse models [23]. It has been observed that the gut microbiota of obese mice exhibited an enhanced capability to harvest energy from food. Specifically, Bacillota dominance was noted in obese mice, whereas Bacteroidota dominated in lean mice, a characteristic also observed in our previous animal study [18]. The BN-202M group showed a statistically significant decrease in body fat percentage compared to the placebo group; therefore, we analyzed the changes in the gut microbiota in the two groups. However, no significant differences in the alpha diversity or taxonomic composition of the gut microbiota were observed between the BN-202M and placebo groups. Further investigation was conducted to compare the participants of the top and bottom 25% body fat reduction rates at baseline and 12 weeks following BN-202M intake. The top 25% of the BN-202M group exhibited a statistically significant increase in Akkermansia compared to the bottom 25% of the BN-202M group. Akkermansia, recognized as a next-generation probiotic [24,25], exhibits a propensity to proliferate post-probiotic consumption but shows reduced levels in obese individuals compared to those with normal weight [26]. Evidence from a longitudinal study indicates that successful weight reduction among obese individuals coincides with heightened Akkermansia levels in fecal samples [27]. Concurrently, our findings consistently revealed a marked negative correlation with Akkermansia in the bottom 25% of the BN-202M group. Thus, this microorganism merits further investigation to explore its association with obesity.

Although the taxonomic composition was not clearly different between the top and bottom 25% of body fat reduction rates at both the baseline and following 12 weeks of BN-202M intake, obvious metabolome differences were observed among the groups. Notably, decreasing patterns of β-alanine and glutamic acid were observed in the top 25% of the BN-202M intake cohort, as was consistently observed in mouse feces in our previous study [16]. Notably, 3-aminoisobutyric acid, suggested as an important metabolite of obesity, decreased in the feces of the BN-202M-supplemented group [28]. Additionally, a diminished pattern of octopamine was observed in the top 25% of the BN-202M group [29]. Thus, we hypothesized that these four metabolites could be potential biomarkers indicating the efficacy of BN-202M supplementation.

This clinical trial has some limitations. This study focused on pre-obese participants with a limited sample size, making it difficult to extrapolate these findings to a broader population. This study focused on overweight adults with BMI measurements ranging from 25 to 29.9, presenting challenges in identifying and generating statistically significant data through clinical trial results. For more robust statistical outcomes, future studies should focus on individuals with obesity. Although DEXA was employed to enhance the precision of the body composition measurements, it only assessed the overall body composition. For future research, using CT scans to observe the reduction in visceral and subcutaneous fat may help demonstrate the improvement in body fat composition. Even though there are limitations, the results show clinically significant effects on body fat reduction, supported by metabolomic and microbiome data, suggesting that these probiotics can be used to induce body fat reduction in obese individuals.

## 5. Conclusions

There was a statistically significant decrease in body fat percentage and body fat mass measured using DEXA in the BN-202M group compared to those in the placebo group without adverse reactions. In terms of body weight and BMI reduction, which are important factors for reducing body fat, a statistically significant change in weight loss and BMI reduction was observed only in the BN-202M group. The probiotic combination BN-202M was effective in reducing body fat, elucidating its mechanism through metabolomic and gut microbiota changes, and confirming its efficacy and safety with human clinical data.

## Figures and Tables

**Table 1:** Baseline characteristics of the participants.

Characteristics	BN-202M (*n* = 67)	Placebo (*n* = 58)	*p*-Value
Sex			0.183 ^1^
Male	8 (11.94)	12 (20.69)	
Female	59 (88.06)	46 (79.31)	
Age (year)	46.82 ± 7.66	44.45 ± 7.93	0.046 ^2^
20–29 (n (%))	2 (2.99)	3 (5.17)	
30–39 (n (%))	8 (11.94)	7 (12.07)	
40–49 (n (%))	27 (40.30)	32 (55.17)	
50–59 (n (%))	30 (44.78)	16 (27.59)	
Height (cm)	160.44 ± 6.83	162.81 ± 7.41	0.103 ^2^
Weight (kg)	69.87 ± 6.96	71.96 ± 7.08	0.053 ^2^
BMI (kg/m^2^)	27.09 ± 1.39	27.11 ± 1.33	0.888 ^2^
SBP (mmHg)	124.87 ± 8.21	124.36 ± 7.67	0.525 ^2^
DBP(mmHg)	77.65 ± 8.95	77.19 ± 9.40	0.823 ^2^
Pulse (beats/min)	81.77 ± 8.30	79.68 ± 9.71	0.361 ^2^
Body temperature (°C)	36.46 ± 0.13	36.47 ± 0.11	0.509 ^2^
Currently an alcohol drinker			0.913 ^1^
Yes	33 (49.25)	28 (48.28)	
No	34 (50.75)	30 (51.72)	

The values are presented as the number (%) or mean ± SD. ^1^ *p* value, estimated using Pearson’s chi-square test; ^2^ *p* value, estimated using the Mann–Whitney U test. Statistical significance was set at *p* <0.05. BMI, body mass index; SBP, systolic blood pressure; DBP, diastolic blood pressure.

**Table 2:** Anthropometric measurements and biochemical variables in blood at baseline and 12 weeks.

Variables	BN-202M (*n* = 67)			*p*-Value	Placebo (*n* = 58)			*p*-Value	BN-202M vs. Placebo *p*-Value
	Baseline	12 Weeks	Mean Change		Baseline	12 Weeks	Mean Change		
Anthropometric measurements									
Body fat (%)	39.62 ± 4.74	39.52 ± 4.71	−0.10 ±1.32	0.557 ^3^	38.06 ± 5.13	38.54 ±5.34	0.48 ± 1.10	0.001 ^3^	0.009 ^1^
Body fat mass (kg)	26.43 ± 3.46	26.19 ± 3.35	−0.24 ± 1.19	0.111 ^3^	26.15 ± 3.43	26.38 ± 3.60	0.23 ± 1.05	0.098 ^3^	0.023 ^1^
Lean body mass (kg)	42.82 ± 6.43	42.63 ± 6.62	−0.19 ± 1.11	0.160 ^3^	45.36 ± 7.16	44.85 ± 7.25	−0.52 ± 0.97	<0.001 ^3^	0.088 ^1^
Body weight (kg)	69.87 ± 6.96	69.29 ± 7.17	−0.58 ± 1.59	0.004 ^3^	71.96 ± 7.08	71.63 ± 7.08	−0.33 ± 1.32	0.060 ^3^	0.352 ^1^
BMI (kg/m^2^)	27.09 ± 1.39	26.87 ± 1.50	−0.23 ± 0.60	0.003 ^3^	27.11 ± 1.33	26.99 ± 1.50	−0.11 ± 0.52	0.099 ^3^	0.263 ^1^
Waist circumference (cm)	88.21 ± 6.22	87.98 ±6.45	−0.22 ± 0.97	0.064 ^3^	88.15 ± 7.24	88.01 ± 7.41	−0.13 ± 0.82	0.217 ^3^	0.583 ^1^
Hip circumference (cm)	101.86 ± 4.77	101.63 ± 4.89	−0.23 ± 0.92	0.013 ^4^	103.33 ± 5.13	103.11 ± 5.06	−0.22 ± 1.50	0.169 ^4^	0.408 ^1^
Waist-to-hip ratio	0.87 ± 0.05	0.87 ± 0.05	0.00 ± 0.00	0.455 ^4^	0.85 ± 0.06	0.85 ± 0.06	−0.00 ± 0.01	0.545 ^4^	0.229 ^2^
Biochemical variables									
Glucose (mg/dL)	91.84 ± 10.28	90.37 ± 10.20	−1.46 ± 9.24	0.200 ^3^	93.47 ± 12.67	92.74 ± 11.23	−0.72 ± 11.94	0.286 ^4^	0.780 ^2^
Total cholesterol (mg/dL)	208.85 ± 35.88	208.07 ± 39.38	−0.78 ± 26.21	0.809 ^3^	201.72 ± 36.39	205.26 ± 34.95	3.53 ± 22.67	0.240 ^3^	0.331 ^1^
HDL cholesterol (mg/dL)	60.52 ± 12.03	58.85 ± 13.01	−1.67 ± 9.41	0.070 ^4^	57.50 ± 14.31	56.33 ± 12.97	−1.17 ±6.53	0.177 ^3^	0.592 ^2^
LDL cholesterol (mg/dL)	126.91 ± 30.54	131.28 ± 31.50	4.37 ± 21.71	0.104 ^3^	122.57 ± 32.87	129.71 ± 35.05	7.14 ± 19.40	0.007 ^3^	0.457 ^1^
Triglyceride (mg/dL)	127.22 ± 60.47	122.03 ± 56.57	−5.19 ± 46.12	0.360 ^3^	131.50 ± 68.46	136.47 ± 74.58	4.97 ± 61.26	0.540 ^3^	0.303 ^1^
Adiponectin (ng/mL)	7921.10 ± 4408.74	7773.28 ± 4807.92	−147.82 ± 2123.23	0.109 ^3^	7482.82 ± 4831.03	7332.55 ± 4333.95	−150.27 ± 1584.76	0.378 ^3^	0.319 ^2^
Leptin (ng/mL)	24.02 ± 10.88	26.69 ± 15.30	2.66 ± 10.18	0.028 ^4^	26.50 ± 15.71	26.58 ± 16.01	0.08 ± 10.80	0.955 ^3^	0.204 ^2^
Insulin (ulU/mL)	7.56 ± 9.66	5.70 ± 3.45	−1.87 ± 10.37	0.131 ^4^	7.54 ± 3.88	7.06 ± 4.99	−0.48 ± 5.73	0.020 ^4^	0.443 ^2^

The values are presented as the mean ± SD. ^1^ *p* value, estimated using the independent two-sample t-test. ^2^ *p* value, estimated using the Mann–Whitney U test. ^3^ *p* value, estimated using the paired *t*-test. ^4^ *p* value, estimated using the Wilcoxon signed-rank test. Statistical significance was set at *p* <0.05. BMI, body mass index; HDL, high-density lipoprotein; LDL, low-density lipoprotein.

**Table 3:** Blood biochemistry at each point.

Variables	Treatment (*n* = 75)			Placebo (*n* = 74)		
	Baseline	6 Weeks	12 Weeks	Baseline	6 Weeks	12 Weeks
Hematological						
WBC (10³/μL)	6.17 ± 1.56	6.30 ± 1.54	6.24 ± 1.61	6.18 ± 1.42	6.05 ± 1.61	5.89 ± 1.64
RBC (10^6^/μL)	4.87 ± 0.41	4.79 ± 0.42 ^3^	4.77 ± 0.41 ^2^	4.89 ± 0.42	4.85 ± 0.39	4.85 ± 0.43
Hemoglobin(g/dL)	14.51 ± 1.66	14.32 ± 1.63 ^3^	14.18 ± 1.52 ^3^	14.32 ± 1.57	14.17 ± 1.44 ^2^	14.17 ± 1.51 ^3^
Hematocrit (%)	43.14 ± 4.20	42.60 ± 4.12	42.31 ± 3.93 ^2^	42.74 ± 3.91	42.42 ± 3.61	42.42 ± 3.94
Platelets (10³/μL)	310.35 ± 69.46	303.75 ± 66.83 ^1^	309.77 ± 75.25	305.69 ± 73.41	312.36 ± 71.27	305.01 ± 64.14
Hematochemistry						
Albumin (g/dL)	4.32 ± 0.30	4.30 ± 0.25	4.31 ± 0.21	4.34 ± 0.29	4.33 ± 0.24	4.32 ± 0.23
ALT (IU/L)	21.40 ± 10.08	20.43 ± 10.87	21.39 ± 13.54	22.93 ± 10.76	22.26 ± 15.03 ^3^	21.15 ± 13.15 ^3^
AST (IU/L)	22.91 ± 6.66	21.79 ± 6.81	23.09 ± 7.45	22.55 ± 6.85	23.90 ± 17.79	22.79 ± 10.53
γ-GT (IU/L)	27.80 ± 26.48	27.79 ± 26.89	26.48 ± 24.51	28.26 ± 29.40	25.90 ± 26.65	26.88 ± 28.87
ALP (IU/L)	62.15 ± 23.30	60.17 ± 22.50 ^3^	62.00 ± 22.89	62.69 ± 16.22	58.35 ± 14.33 ^2^	59.14 ± 13.80 ^2^
BUN (mg/dL)	13.36 ± 3.88	12.80 ± 4.13	12.65 ± 3.52	12.63 ± 3.63	12.74 ± 3.47	12.51 ± 3.10
Creatinine (mg/dL)	0.78 ± 0.13	0.78 ± 0.15	0.77 ± 0.14	0.78 ± 0.15	0.78 ± 0.14	0.78 ± 0.14
Glucose (mg/dL)	92.19 ± 10.22	91.59 ± 13.45	90.85 ± 11.11	94.01 ± 12.94	90.88 ± 13.09	93.27 ± 11.97
Total bilirubin (mg/dL)	0.80 ± 0.32	0.76 ± 0.29	0.72 ± 0.32 ^3^	0.73 ± 0.28	0.74 ± 0.28	0.72 ± 0.33
Triglyceride (mg/dL)	130.35 ± 67.01	150.35 ± 160.33	132.13 ± 77.51	131.08 ± 0.41	132.79 ± 76.59	133.24 ± 75.26
Total cholesterol (mg/dL)	206.81 ± 36.43	209.95 ± 37.59	208.71 ± 38.66	199.72 ± 38.99	198.90 ± 40.88	202.57 ± 36.64
HDL cholesterol (mg/dL)	59.76 ± 11.81	59.85 ± 13.14	58.49 ± 12.95	57.15 ± 13.79	57.61 ± 13.94	55.94 ± 12.27 ^2^
LDL cholesterol (mg/dL)	125.57 ± 31.11	127.76 ± 28.83	130.64 ± 31.18	121.80 ± 35.74	120.65 ± 36.63	127.42 ± 35.22 ^2^
Uric acid (mg/dL)	5.09 ± 1.09	4.98 ± 1.07 ^1^	4.92 ± 1.11 ^1,2^	5.07 ± 1.29	5.18 ± 131	5.10 ± 1.17

The values are presented as the mean ± SD. ^1^ Estimated using the Mann–Whitney U test. ^2^ Estimated using the paired *t*-test. ^3^ Estimated using the Wilcoxon signed-rank test. WBC, white blood cell; RBC, red blood cell; ALT, alanine aminotransferase; AST, aspartate aminotransferase; γ-GTP, gamma-glutamyl transferase; ALP, alkaline phosphatase; BUN, blood urea nitrogen; HDL, high-density lipoprotein; LDL, low-density lipoprotein.

## Data Availability

The data presented in this study are included in the article. Further inquiries can be directed to the corresponding author.
